# Identification of MicroRNA for Intermuscular Bone Development in Blunt Snout Bream (*Megalobrama amblycephala*)

**DOI:** 10.3390/ijms160510686

**Published:** 2015-05-11

**Authors:** Shi-Ming Wan, Shao-Kui Yi, Jia Zhong, Chun-Hong Nie, Ning-Nan Guan, Bo-Xiang Chen, Ze-Xia Gao

**Affiliations:** 1 College of Fisheries, Key Lab of Agricultural Animal Genetics, Breeding and Reproduction of Ministry of Education/Key Lab of Freshwater Animal Breeding, Ministry of Agriculture, Huazhong Agricultural University, Wuhan 430070, China; E-Mails: wanshiming@webmail.hzau.edu.cn (S.-M.W.); yishaokui@foxmail.com (S.-K.Y.); zhongjia@webmail.hzau.edu.cn (J.Z.); nchunh44@yahoo.com (C.-H.N.); 13007100773@163.com (N.-N.G.); ChenBX@haid.com.cn (B.-X.C.); 2 Freshwater Aquaculture Collaborative Innovation Center of Hubei Province, Wuhan 430070, China; 3 Animal Husbandry and Fisheries Research Center of Haid Group Co., Ltd., Guangzhou 511400, China

**Keywords:** intermuscular bone, teleosts, microRNA, *Megalobrama amblycephala*

## Abstract

Intermuscular bone (IB), which occurs only in the myosepta of the lower teleosts, is attracting more attention of researchers due to its particular development and lack of genetic information. MicroRNAs (miRNAs) are emerging as important regulators for biological processes. In the present study, miRNAs from IBs and connective tissue (CT; encircled IBs) from six-month-old *Megalobrama amblycephala* were characterized and compared. The results revealed the sequences and expression levels of 218 known miRNA genes (belonging to 97 families). Of these miRNAs, 44 known microRNA sequences exhibited significant expression differences between the two libraries, with 24 and 20 differentially-expressed miRNAs exhibiting higher expression in the CT and IBs libraries, respectively. The expressions of 11 miRNAs were selected to validate in nine tissues. Among the high-ranked predicted gene targets, differentiation, cell cycle, metabolism, signal transduction and transcriptional regulation were implicated. The pathway analysis of differentially-expressed miRNAs indicated that they were abundantly involved in regulating the development and differentiation of IBs and CT. This study characterized the miRNA for IBs of teleosts for the first time, which provides an opportunity for further understanding of miRNA function in the regulation of IB development.

## 1. Introduction

Intermuscular bones (IBs), which occur only in lower teleosts amongst vertebrates, are segmental, serially homologous ossifications in the myosepta [1]. They appeared to have a function to strengthen the connection between the sarcomeres [2]. Obviously, the ossification process and distribution of IBs was different from other axial skeletons [3]. The IBs develop directly from mesenchymal condensation, being totally different from the ribs, which are developed from a mesenchymal cell population derived from the ventral somite. The IBs are primordially differentiated from osteoblast in connective tissue, which is essentially different from normal muscle. Most of the freshwater aquaculture fishes around the world, especially Cyprinidae species, such as crucian carp (*Carassius carassius*), grass carp (*Ctenopharyngodon idellus*), silver carp(*Hypophthalmichthys* *molitrix*), *etc.*, possess a certain amount of IBs. These IBs have brought about many adverse impacts on the economic and food value of a species, such as influencing the processing and affecting the freshness [4]. Along with the evolution of fish species, the morphology tends to be more complicated, especially becoming extremely complex with the evolution of the cyprinids [5]. In view of its potential value, researchers began to study IBs in fish as early as the 1960s. Bing [6] first described the morphology of IBs in juvenile common carp (*Cyprinus carpio*). Patterson *et al.* [1] and Johnson *et al.* [7] have made detailed observations and analyses of the IBs for 125 teleostean fish species, including Chinese major freshwater cyprinids. Subsequently, extensive research has been undertaken focusing on the morphology, number and distribution of the IBs in many fish species [8,9,10]. Interestingly, Li *et al.* [11] documented the number of IBs in different ploidies of *C. auratus* and found significant differences among the different ploidy fishes, which indicated the possibility of decreasing the number of IBs through genetic improvement. With the rapid development of molecular breeding technologies, it is possible to suppress IB formation by gene silencing, gene knockout and fluorescent protein methods combined with gene inhibitor technology.

It is well known that miRNA is a special kind of molecule in organisms, which regulates the level of proteins by decreasing messenger RNA (mRNA) levels or inhibiting translation by binding the 3' UTR of the target mRNA. miRNA plays an important role in various developmental, physiological and pathological conditions, such as osteoblast differentiation, development, disease, gene transcription and translation [12]. The description of miRNA has been recorded for several fish species, such as Atlantic salmon (*Salmo salar*), medaka (*Oryzias latipes*), rainbow trout (*Oncorhynchus mykiss*), *C. carpio*, bighead carp (*Hypophthalmichthys nobilis*), *H. molitrix*, channel catfish (*Ictalurus punctatus*) and Nile tilapia (*Oreochromis niloticus*). The progress of these studies in fish provides insight into the types of miRNAs and their possible mechanisms in sexual development [13], evolution evaluation [14], skeletal muscle development [15] and growth [16]. Nonetheless, few studies have been conducted on the identification of miRNA for fish IBs.

Blunt snout bream (*Megalobrama amblycephala*, Yih, 1955), which is restrictively distributed in the middle and lower reaches of the Yangtze River in Central China, is a fish species with high economic value, which has been farmed in China’s freshwater polyculture systems since the 1960s [17]. Intensive studies of this species have been performed, especially concerning growth, disease control and genetic resources [18,19,20]. Our previous study has documented the emergent periods and morphogenesis of IBs in *M. amblycephala* [21]. In the present study, we implemented an miRNA comparative analysis to investigate the miRNAs expression and regulated pattern of IBs and connective tissue (CT), which encircle IBs, through high throughput sequencing technology. The miRNAs in two tissues of *M. amblycephala* were identified, and the differentially-expressed miRNAs were analyzed. The obtained miRNA resources from this study will contribute to a further understanding of the molecular mechanisms of IBs development and the roles that miRNAs play in regulating diverse biological processes in fish.

## 2. Results and Discussion

### 2.1. General Features of Small RNAs

**Figure 1:**
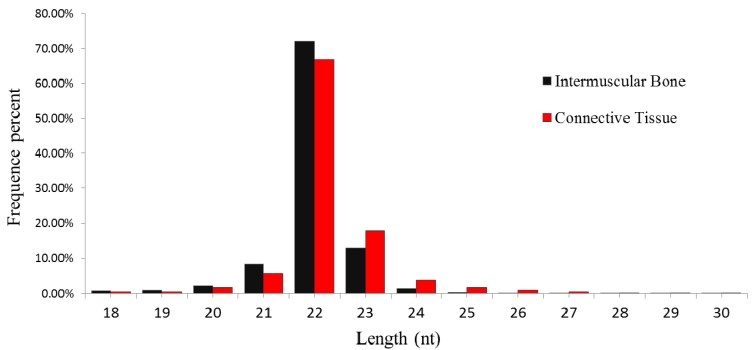
Length distribution of small RNAs in intermuscular bone and connection tissue of *M. amblycephala*.

In order to identify the miRNAs for IBs of *M. amblycephala*, the two tissues (IBs and CT, which encircle the IBs) were collected from six-month-old individuals, when the intermuscular bone is actively growing, based on our previously published study [21]. Two independent RNA libraries were constructed for the IBs and CT and then sequenced using the Illumina HiSeq 2000 platform. A total of 9,544,686 and 9,375,253 raw reads were generated from the IB and CT libraries, respectively. After discarding low-quality reads, 3' and 5' adaptors, sequences with <18 and >30 nt, 9,424,634 (99.07%) and 9,211,591 (98.52%) clean small RNA reads were obtained in the IB and CT libraries, respectively (Table S1-1). Little difference was found in the length distribution of the sequences from the two libraries. Most (>93%) of the small RNAs were 21–23 nt in length, especially 22 nt, which is the typical length of Dicer-derived products, accounting for 64.29% and 72.01% of the total sequence reads in the IB and CT libraries, respectively (Figure 1). These results are similar to the length distribution characteristics of small RNAs in other fish species, such as *C. carpio* [22], *I. punctatus* [23] and Japanese flounder (*Paralichthys olivaceus*) [24].

**Figure 2:**
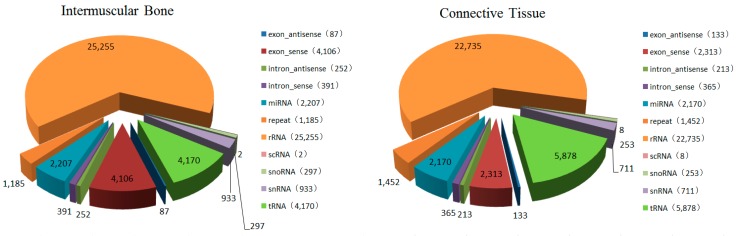
Annotation of small RNAs derived from the Intermuscular bones (IBs) and connective tissue (CT) of *M. amblycephala*.

The total number of unique reads from sRNA libraries of IB and CT groups was 168,227 and 249,063, respectively. These unique sequences of two libraries contained 34,314 common sequences (Table S1 and Table S2). After comparing the small RNA sequences with the NCBI GenBank and Rfam database, rRNA, tRNA, snRNA, snoRNA, scRNA, repeat DNA, exon antisense, exon sense, intron antisense and intron sense sequences were annotated and removed (Figure 2). The remaining reads, including 9,152,701 and 8,884,176 for the IB and CT groups, respectively, consisting of 131,549 and 215,002 unique sequences, respectively (Table S1-3), were retained for miRNA analysis.

Due to the lack of genome information for *M. amblycephala*, the selected small RNA sequences were mapped to the genome sequence of zebrafish (*Danio rerio*), which is evolutionarily close to *M. amblycephala*. For the selection of the computing algorithm, we chose a tolerance of one mismatch for mapping. Subsequently, for the IB and CT groups, 8,389,883 reads (89.02%) representing 69,307 (41.2%) unique small RNAs and 7,534,341 reads (81.79%) representing 48,844 (19.61%) unique small RNAs, respectively, were mapped to the reference genome (Figure S1).

### 2.2. Identification of Conserved miRNAs

To identify conserved miRNAs, the sequences of the miRNAs in the libraries of the IB and CT groups were compared with those of the 1250 precursor miRNAs and 831 mature miRNAs from miRBase Version 20.0. This analysis identified 201 and 205 conserved miRNAs in the IBs and CT libraries, respectively. The reads of these miRNAs were ranged from 1 to 2,796,874, indicating that not only highly-expressed miRNAs, but also weakly-expressed miRNAs were identified by Illumina small RNA deep sequencing. After grouping identical sequences, a total of 218 unique mature miRNAs were identified, which belong to 97 families, including 188 miRNAs that overlapped between the two libraries, 13 and 17 miRNAs that were detected only in the IB and CT libraries, respectively (Table S2).

For the identified and validated miRNAs from two groups, the ten most abundant miRNAs in the IBs were miR-1, let-7a, miR-206, let-7b, let-7c, miR-199-3p, miR-21, let-7f, let-7d and miR-199a-3p, accounting for 94.86% of the total reads mapped to miRBase. Eight of these miRNAs were also among the ten most abundant miRNAs (miR-1, miR-206, let-7a, let-7b, let-7c, miR-199-3p, miR-21, miR-22a, let-7f, let-7g) identified in the CT library, which accounted for 94.28% of the total reads mapped to miRBase. For these miRNAs that have already been identified and validated, miR-1 has the highest expression in both libraries. This phenomenon was in agreement with other studies of miRNAs [15,25,26], showing that miR-1 was the crucially expressed miRNAs in skeletal muscle development, human osteosarcoma origin, proliferation and cell cycle control. miR-206, previously viewed as a muscle-specific miRNA [27,28], was demonstrated as a key regulator for the process of osteoblast differentiation. The expression of miR-206 decreased over the course of osteoblast differentiation, and overexpression of miR-206 in osteoblasts inhibited their differentiation; conversely, knockdown of miR-206 expression promoted osteoblast differentiation [29]. Therefore, we speculated that the high expression of miR-206 in IB and CT libraries could be related to the development and differentiation of IBs. Previous studies have shown that the target genes of identified miRNAs could be involved in the basic biological reactions and organ system development. For instance, miR-21 contributes to myocardial disease by stimulating MAP kinase signaling in fibroblasts [30], and it was known that downregulation of miR-21 biogenesis by estrogen action results in osteoclastic apoptosis [31]. A previous study also found that miR-22 regulated the adipogenic and osteogenic differentiation of human adipose tissue-derived mesenchymal stem cells in opposite directions, which indicated that miR-22 was decreased during the process of adipogenic differentiation, but increased during osteogenic differentiation [32]. Similarly, miR-199a-3p had been also reported to play a functional role in osteosarcoma cell growth and proliferation [33]. Transfection of precursor miR-199a-3p into osteosarcoma cell lines significantly decreased cell growth and migration. Restoring miR-199a-3p’s function may provide therapeutic benefits in osteosarcoma [33]. These findings may be able to assist us better in explaining the development mechanism of IBs. In addition, seven miRNAs of the let-7 family, including let-7a, let-7b, let-7c, let-7d, let-7e, let-7f and let-7g, were present at high frequencies in both libraries. This result was similar to many previous studies about miRNAs in fish species [24,25,34,35,36], which demonstrated that the let-7 miRNAs are important regulators for extensive biological processes. Interestingly, the expression of let-7d had a significant difference in IBs and CT. Huleihel and Ben-Yehudah [37] had examined the effects of transfection on fibroblast responsiveness to transforming growth factor-β (TGF-β) and found that let-7d transfection significantly attenuated high-mobility protein induction by TGF-β. Notably, TGF-β was an important factor in osteoblast differentiation and bone formation [38]. Therefore, we conjectured that let-7d may play a key role in the differentiation process between IBs and CT through regulating protein synthesis of osteoblast differentiation and bone formation.

Additionally, the analysis of base bias on the first position of identified miRNAs with a certain length and on each position of all identified miRNA was performed, respectively. Results showed that the identified sequences being 18–25 nt in length from two libraries have a strong bias for U in the first nucleotide (Figure S2 and Figure S3). Furthermore, a total of 25 duplex-like miRNA:miRNA* pairs were detected from 4377 unique sequences. It had been reported that miRNA/miRNA* ratios may vary dramatically at different stages of development, as some miRNA* sequences could be reported as mature functional miRNAs with abundant expression [24]. In our study, we also found that some miRNA*s were detected at obviously high levels in two libraries, like miR-199-3p, miR-199a-3p, miR-140-3p and mam-miR-133a-3p (Table S2). Previous studies [39,40] have described the same phenomenon and indicated that the miRNA*s may play a functional role in regulating gene expression.

### 2.3. Statistics of Multiple IsomiRs in M. amblycephala

**Figure 3:**
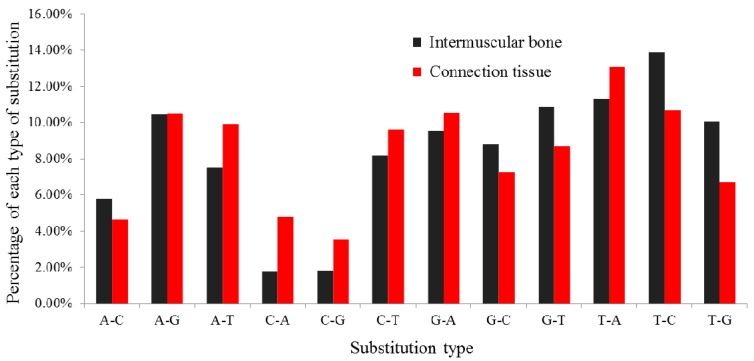
Histogram displaying the single nucleotide substitutions in the miRNAs seed region sequence when aligning un-annotated sRNAs tags with mature miRNAs from miRBase20.0. The *x*-axis represents the substitution type from genome to RNA (small RNA sequence). The *y*-axis represents the percentage comparing of the observed count of each type to the total count of all substitution types.

Recently, deep sequencing revealed that the same miRNA precursor can generate multiple isoforms of miRNA (isomiRs), which vary in length and/or sequence, due to exonuclease-mediated trimming, a shift of cleavage sites of Drosha and Dicer, miRNA editing or 3'-end nontemplated nucleotide addition. In the present study, the phenomenon of multiple isomiRs was again observed. Some isomiRs, detected with single nucleotide substitutions, including transition and transversion, possibly represent the result of pre-miRNA editing. These end-sequence variations are interesting, as they may allow miRNA variants to perform distinct roles through influencing the formation of the miRNA/target mRNA hybrid duplex. Of the twelve types of single nucleotide substitutions observed in the two libraries, the most prominent were T to A (12.50%), T to C (11.76%), A to G (10.51%) and G to A (10.21%) (Figure 3), which was similar to several previous studies [16,24,41]. Meanwhile, prior reports had demonstrated that sequence variations may be the result of post-transcriptional modifications of RNA [42]. Therefore, we presume that the nucleotide substitutions in this study may be attributed to post-transcriptional modifications of RNA.

**Figure 4:**
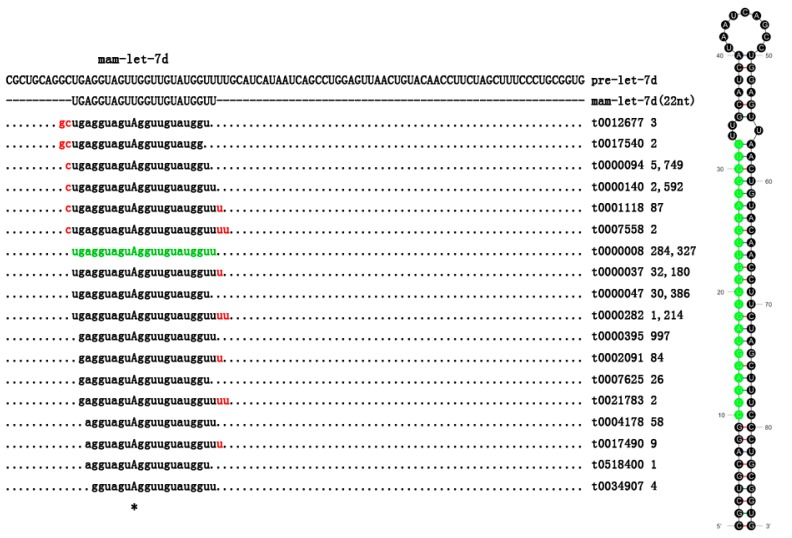
A portion of the miRNA precursor and details of mam-let-7d isomiRs, including sequence count. The most abundant mature miRNAs are indicated by the sequence in green. The non-templated nucleotide additions were indicated in the red. * indicated the single nucleotide mutation loci.

Furthermore, another type of isomiR was observed with additional 5' or 3' non-template nucleotides, which may have longer, shorter or consensus lengths considering the canonical miRNA sequences [42,43]. The most abundant nontemplated nucleotide added at the 3' terminal ends of mature miRNA were uridine. A typical example was mam-let-7d-3p, in which the length varied from 19–25 nucleotides, similar to other miRNAs identified in this study (Figure 4). A similar phenomenon has previously been widely reported in many species included fish [16,23], maize [44] and mouse [45], showing that essentially, all miRNAs have length and/or end-sequence variation. Different from the nucleotide substitutions, these length variations were thought to be the result of inexact Drosha and Dicer processing. Additional 3' non-template nucleotides in isomiRs may play a key role in miRNA:target interactions. IsomiRs with 3' additions increased miRNA stability in *Drosophila* and weakened the effectiveness of some specific miRNAs, and isomiRs could also accumulate to a considerable level and downregulate their target genes in organisms [46,47,48,49]. Summarily, more future studies are required to functionally validate the conclusion and the significance of isomiRs.

### 2.4. Differentially Expressed miRNAs

**Figure 5:**
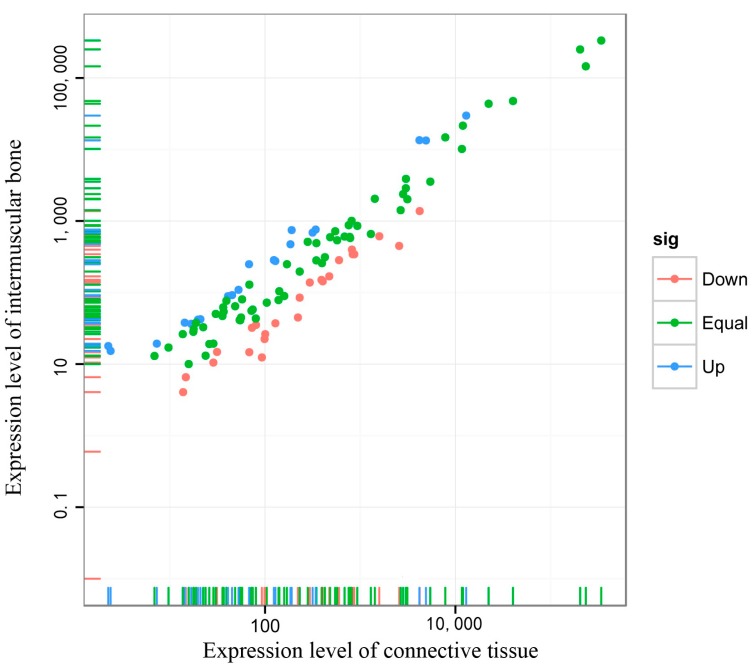
Scatter plot map for miRNA expression levels in the IBs and CT of *M. amblycephala*. Each plot represents an individual miRNA. This reflects the proportion of miRNAs that have a greater number in IBs and CT, respectively.

Among these conserved 218 miRNAs identified by RNA-Seq, 44 conserved miRNAs in *M. amblycephala* were differentially expressed (*p* < 0.01) by comparing with miRNA expression patterns between two libraries (Table S3). Of the 44 differentially-expressed miRNAs, 24 miRNAs and 20 miRNAs had higher expression in the CT and IB groups, respectively, as shown by differential expression analysis, which suggested that miRNAs play an important role in regulating diverse biological processes during the development of IBs. The expression of miRNA in two samples was shown by plotting a log2-ratio figure and scatter plot (Figure 5). These differentially-expressed miRNAs were sequenced at varying frequencies. For instance, mam-miR-199-3p, mam-miR-199a-3p, mam-miR-128 and mam-let-7d were detected with relatively high sequence counts in both libraries. In contrast, the sequencing frequencies of some miRNAs (mam-miR-363, mam-miR-30b and mam-miR-551) were low in both of the libraries.

**Figure 6:**
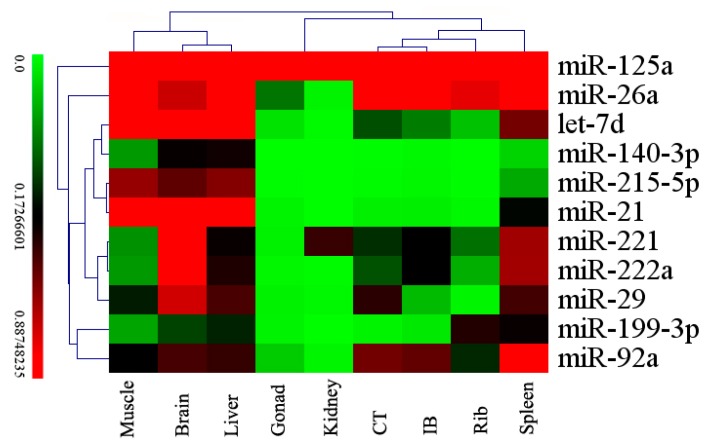
Heat map showing the 11 differentially-expressed miRNAs expression patterns in nine tissues measured by stem-loop RT-PCR. Relative expression levels of the 11 differentially-expressed miRNAs were measured in terms of threshold cycle value (*C*_t_) and were normalized to 5S rRNA. The expression data were analyzed by hierarchical clustering for both tissues and miRNAs.

Quantifying the differentially expressed miRNAs in the different tissues is an important initial step to investigate the fundamental functions of these miRNAs. We used stem-loop RT-PCR to validate and profile the expression of the 11 differentially-expressed miRNAs in nine tissues of *M. amblycephala*, including muscle, brain, liver, spleen, kidney, gonad, rib, IBs and CT (Figure 6). The results indicated that the mam-miR-221, mam-miR-222a, mam-miR-92a and mam-miR-26a were expressed at relatively high levels in IBs and CT, and this result suggests that these miRNAs may play roles in the connective tissue differentiation and IB formation of *M. amblycephala*. Simultaneously, we found the expression of mam-miR-125a and -26a was almost significantly high in all tissues. Ubiquitous expression of these miRNAs indicates that they were involved in many fundamental functions. For example, mam-miR-125a not only affected genes involved in the mitogen-activated protein kinases (MAPKs) signaling pathway [50], but also may act as an NF-κB inhibitor upon TLR stimulation and inhibits erythroid differentiation in leukemia and myelodysplastic syndromes(MDS) cell lines [51].

### 2.5. Prediction of Potential Targets of Differentially-Expressed miRNA

The identification of miRNA targets is a crucial step to further understand the regulatory functions of miRNAs. Strong base pairing between the seed region of a miRNA and the UTR of its target mRNA is important for its degree of regulation. Hence, many target prediction algorithms enforce strong seed complementarity and evolutionary conservation in the complementary region of potential targets. As such, to improve the accuracy of target gene prediction results and to limit the validation requirements, targets were identified by both MiRanda [52] and TargetScan [53] and were present in the results of comparative transcriptome sequencing analysis. A total of 2,084,911 unique targets, including 49,962 EST sequences of *M. amblycephala*, were predicted for the 44 highly significant differently-expressed miRNAs (*p* < 0.01), which were identified in this study. The predicted targets for the differently-expressed miRNAs are shown in Table S4.

### 2.6. Function Analysis of Target Genes of Differentially-Expressed miRNAs

The identified target genes of differentially-expressed miRNAs were subjected to a GO analysis, which classifies miRNA-gene regulatory networks on the basis of molecular function, cellular component and biological process. The GO analysis of identified predicted target genes revealed 21,082, 20,087 and 20,627 genes, respectively, classified into 877 molecular function ontology terms, 388 cellular component ontology terms and 3633 biological process ontology terms (Table S5). It was noteworthy that the GO terms of miRNAs between IBs and CT were highly present in: cell 83.60%, intracellular 74.10% and organelle 61.00% of the cellular component; cellular processes 75.70%, single-organism processes 57.40%, metabolic processes 55.90% of biological processes; and binding 76.40%, catalytic activity 44.70% and organic cyclic compound binding 31.80% of molecular functions. The detailed information about the functional classification is shown in Table S6.

The pathways of predicted target genes, which were actively regulated by miRNA in *M. amblycephala*, were also identified and classified according to KEGG functional annotations. Notably, the most overrepresented miRNA targets belonged to the metabolic pathways, which perform a variety of anabolic and catabolic tasks, such as lipid, carbohydrate, amino acid and energy metabolism. A similar phenomenon has previously been widely reported in many studies [54,55], showing that the pathways serve energy conversion, macromolecular compounds synthesis, and so on. The Wnt signaling pathway (2.14%) had previously emerged as a key regulator of developmental processes, including skeletal patterning. Mouse genetics confirmed the importance of canonical Wnt signaling in the regulation of bone homeostasis. Activation and inhibition of the pathway, respectively, lead to increased and decreased bone mass and strength [56]. The importance of Wnt signaling for bone has also been highlighted since then in the general population in numerous genome-wide association studies [57]. TGF-β regulates a large variety of cellular activities, which acts as a potent inhibitor of the terminal differentiation of epiphyseal growth plate chondrocytes during the formation of endochondral bone [58]. In our study, 424 (1.46%) target genes of the differential miRNAs were mapped to the TGF-β signaling pathway. Another pathway targeted by the differential miRNAs was the MAPK signaling pathway (3.17%), which is not only known to be involved in the regulation of muscle differentiation by affecting the activities of myogenic transcription factors, as well as controlling the expression of structural muscle genes [59], but also essential for skeletogenesis and bone homeostasis. In mice, the p38 MAPK pathway is necessary for normal skeletogenesis, and deletion of any of the MAPK pathway member-encoding genes, MAPK kinase 3 (Mkk3), Mkk6, p38a or p38b, profoundly reduced bone mass secondary to defective osteoblast differentiation [60]. In fish, the activation of MAPK is absolutely indispensable for muscle cell proliferation [61]. Meanwhile, 419 (1.45%) target genes of the differential miRNAs were directly mapped to the osteoclast differentiation pathway. In addition, the pathway data also highlighted associated with human T-lymphotropic virus type 1(HTLV-I) infection, dilated cardiomyopathy, Epstein-Barr virus infection, herpes simplex infection and other diseases, suggesting that genes involved in cellular immune, cell cycle progression and cell proliferation are targeted by the differential miRNAs. Furthermore, pathways associated with the regulation of actin cytoskeleton, focal adhesion, biosynthesis of secondary metabolites, endocytosis, phagosome, tight junction, vascular smooth muscle contraction and protein processing in endoplasmic reticulum were all significantly enriched, indicating the role of the differentially-expressed miRNAs in the regulation of cell motility, cytoskeleton, cell nutrition, communication between cells and the extracellular matrix.

On the whole, the results indicated that these differentially-expressed miRNAs were abundant and functionally involved in regulating the development and differentiation of IBs and CT, as well as providing an opportunity for further functional validation of miRNA in the regulation of IB development.

## 3. Experimental Section

### 3.1. Animals and Tissue Collection

All animals and experiments were conducted in accordance with the “Guidelines for Experimental Animals” of the Ministry of Science and Technology (Beijing, China). The study was approved by the Institutional Animal Care and Use Ethics Committee of Huazhong Agricultural University. All efforts were made to minimize suffering.

The experimental animals were collected from an *M. amblycephala* selective population, which were bred in the Ezhou Fish Breeding Base of Huazhong Agricultural University (30°22'N, 114°47'E). All experimental procedures involving fish were approved by the Institution Animal Care and Use Committee of the Huazhong Agricultural University.

The IB and CT samples for small RNA sequencing were collected from six individuals at 6 months old from the same population. The maturation age of this species is normally 2–3 years, and the fish are actively growing at 6 months old. The fish were anaesthetized in well-aerated water containing the 100 mg/L concentration of tricaine methanesulfonate (MS-222) before tissue collection. The IBs and CT, which surround the IBs, were immediately collected to extract total RNA. Other tissue samples, including rib, muscle, liver, kidney, spleen, gonad and brain, were also snap-frozen in liquid nitrogen and stored at −80 °C for total RNA extraction.

### 3.2. Small RNA Isolation and cDNA Library Construction

Total RNA was isolated from the connective tissue using RNAiso Plus Reagent (TaKaRa, Dalian, China), according to the manufacturer’s protocol. The total RNA of IBs was isolated using an innovative method (Chinese Patent No. 201310673534). Briefly, the samples of IBs were separated quickly from muscle tissues and homogenized by grinding in liquid nitrogen. Hydrosaline solution (0.8 M sodium citrate and 1.2M sodium chloride) was used for removing the protein pollution with isopropanol treatment. The total RNA pellet was washed twice with 75% ethanol by vortexing and centrifuging for 5 min at 7500× *g* and then dissolved in 30 μL of RNase-free water. RNA quality and quantity was measured using the NanoDrop 2000 (Thermo Scientific, Waltham, MA, USA) and Agilent 2100 Bioanalyzer (Agilent, Santa Clara, CA, USA). All of the samples were standardized to 500 ng/μL, and equal volumes of the same tissues from different individuals were combined into one pool.

For the construction of two small RNA (sRNA) libraries, small RNAs of 18–30 nt in length were first isolated from the total RNA by size fractionation in a polyacrylamide gel, and these small RNAs were ligated with 5'-RNA and 3'-RNA adapters. Afterwards, reverse transcription PCR was used to create cDNA constructs based on two adapters. The generated small cDNA libraries were amplified by PCR using primers complementary to the adaptor sequences. Subsequently, the amplified cDNA constructs were tested by the Agilent 2100 Bioanalyzer and ABI StepOnePlus Real-Time PCR System and sequenced by Illumina technology (BGI, Shenzhen, China).

### 3.3. Small RNA Sequence Analysis

The sequencing of cDNA libraries was based on the criteria of HiSeq 2000 SE50. By the base calling step, the basic figure from Illumina small RNA deep sequencing is converted into sequence data (raw reads), which is stored in a FASTQ format, including quality data of the reads. Before accurate analysis, low-quality reads, reads with 5' primer contaminants, reads with 3' primer, reads with polyA and reads shorter than 18 nt were eliminated. After filtering, the statistics of data quality and length distribution were performed. The common and specific reads of two samples were summarized, including the summary of unique and total reads.

Afterwards, a standard bioinformatics analysis of the high-quality sequences was implemented. The high-quality reads were blasted against the Rfam (available online: ftp://sanger.ac.uk/pub/databases/Rfam/) database and the GenBank noncoding RNA database (available online: http://blast.ncbi.nlm.nih.gov/) to annotate rRNA, tRNA, snRNA and other ncRNA sequences and aligned to the transcriptome of the *M. amblycephala* (Sequence Read Archive (SRA) Database: SRR1613326, SRR1612557, SRR1613325) to screen and remove the mRNA-derived degraded fragments. The selected sequences were also mapped to the zebrafish genome with a tolerance of one mismatch in the seed sequence to analyze their expression and distribution on the genome by Short Oligonucleotide Analysis Package (SOAP, available online: http://soap.genomics.org.cn/). Subsequently, miRNA identification was performed by comparing the data from two samples with the known mature miRNAs and the miRNA precursor of all fish in miRBase20.0 available online: (http://www.mirbase.org/ftp.shtml). Furthermore, the base bias had been analyzed, respectively, on the first position of identified miRNAs with a different length and on each position of all identified miRNAs. The base edit of known miRNAs (isomiRs) was conducted. The majority of identified miRNAs showed heterogeneity of length and sequence in the prior studies. The nucleotides at position 2–8 of a mature miRNA are known as the seed region, and this region is highly conserved [16,23]. The target of miRNA might be different with the change of nucleotides in this region. In our analysis pipeline, miRNAs, which might have a base edit, can be detected by aligning unannotated sRNA tags with mature miRNAs from miRBase20.0, allowing one mismatch on a certain position.

### 3.4. Differential Expression Analysis of miRNAs

The known miRNA expression level between two samples was compared to find out the differentially-expressed miRNAs. Total miRNA counts were used for normalization, and the miRNAs of reads less than 100 were discarded on account of meaningless biological or technological errors. miRNAs that appeared as differently expressed in two libraries were further analyzed by use of the method described in previous studies [16,62,63]. Briefly, miRNAs expression in two libraries was normalized using the following formula: normalized expression = (actual miRNA sequencing reads count/total clean reads count) × 1,000,000. If the normalized expression (NE) value of a given miRNA were zero, the expression value was modified to 0.01. If the normalized expression of a given miRNA were less than 1 in both libraries, it was removed in future differential expression analyses. The fold-change and *p*-value were calculated from the normalized expression. When |log2ratio| ≥ 1 and *p*-value ≤ 0.05, it was identified as differential expression. A prerequisite for using the data for expression comparisons is that the method applied reproduces the different individual miRNA expression levels in a sample well.

Quantitative stem-loop RT-PCR with SYBR Green PCR Master Mix (TaKaRa, Dalian, China) was performed to profile the expression levels of miRNAs in 9 tissues. Eleven primers for stem-loop RT-PCR were designed according to descriptions in prior study [64] (Table S7). Total RNA from IBs, CT, rib, muscle, brain, liver, spleen, kidney and gonad of *M. amblycephala* was isolated using TRIzol reagent (Invitrogen) following the recommendations of the manufacturer. Real-time PCR was carried out on a Roche LightCycler 480 System II (Roche, Mannheim, Germany) according to the manufacturer’s instructions, and all real-time reactions were performed in triplicate. The primers for stem-loop real-time PCR are shown in Table S7. The relative expression levels of the differentially-expressed miRNAs were measured in terms of threshold cycle value (*C*_t_) and were normalized to 5S rRNA using the equation 2^−∆∆*C*t^, in which ∆*C*_t_ = *C*_t__miRNA_ − *C*_t 5s_.

### 3.5. Prediction of miRNA Target Genes

Considering that genome references of *M. amblycephala* are not available, the sequences of zebrafish genome and transcriptome sequences of *M. amblycephala* sequenced in our laboratory (SRA Database: SRR1613326, SRR1612557, SRR1613325) were selected to predict the target genes. Briefly, miRNAs identified in the present study were used to search for antisense hits in the reference RNA sequences. Subsequently, mRNA sequences exhibiting perfect or near perfect complementarity with corresponding miRNAs were selected and analyzed with TargetScan (available online: http://www.targetscan.org/) and MiRanda (available online: http://www.microrna.org) to predict the target sequences. Furthermore, the biological function of the novel miRNAs was annotated with mapping targets genes to each term of the Gene Ontology database: (available online: http://www.geneontology.org/), and the gene numbers of each GO term were calculated. The main pathways of biochemical and signal transduction significantly associated with the predicted target genes of the miRNAs were determined via a KEGG pathway analysis (available online: http://www.kegg.jp/).

## 4. Conclusions

On the whole, the results indicated that these differentially-expressed miRNAs were abundant and functionally involved in regulating the development and differentiation of intermuscular bone and connective tissue, providing an opportunity to further functionally validate miRNA in the regulation of the development of intermuscular bone.

## Supplementary Materials

All small RNA data are available in the NCBI Gene Expression Omnibus database under Accession SRX868670. The other supporting data are included as supplementary files. Supplementary materials can be found at http://www.mdpi.com/1422-0067/16/05/10686/s1.
