# Supplementary material for: Identification of MicroRNA for Intermuscular Bone Development in Blunt Snout Bream (Megalobrama amblycephala)
Source: Int J Mol Sci. 2015 May 11;16(5):10686–703. doi: 10.3390/ijms160510686 (PMC4463670; doi:10.3390/ijms160510686)
Supplement: Supplementary file 1 [file ijms-16-10686-s001.zip › ijms-78241-SI/ijms-78241-Supplementary Information-Figure S1-S3.pdf]

# Supplementary Information

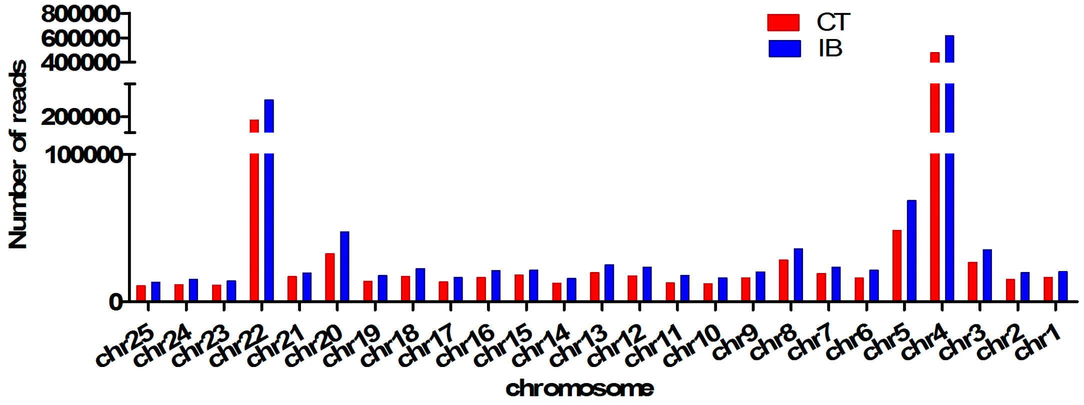


**Figure S1.** Number and distribution of clean reads mapped to the genome sequence of zebrafish.

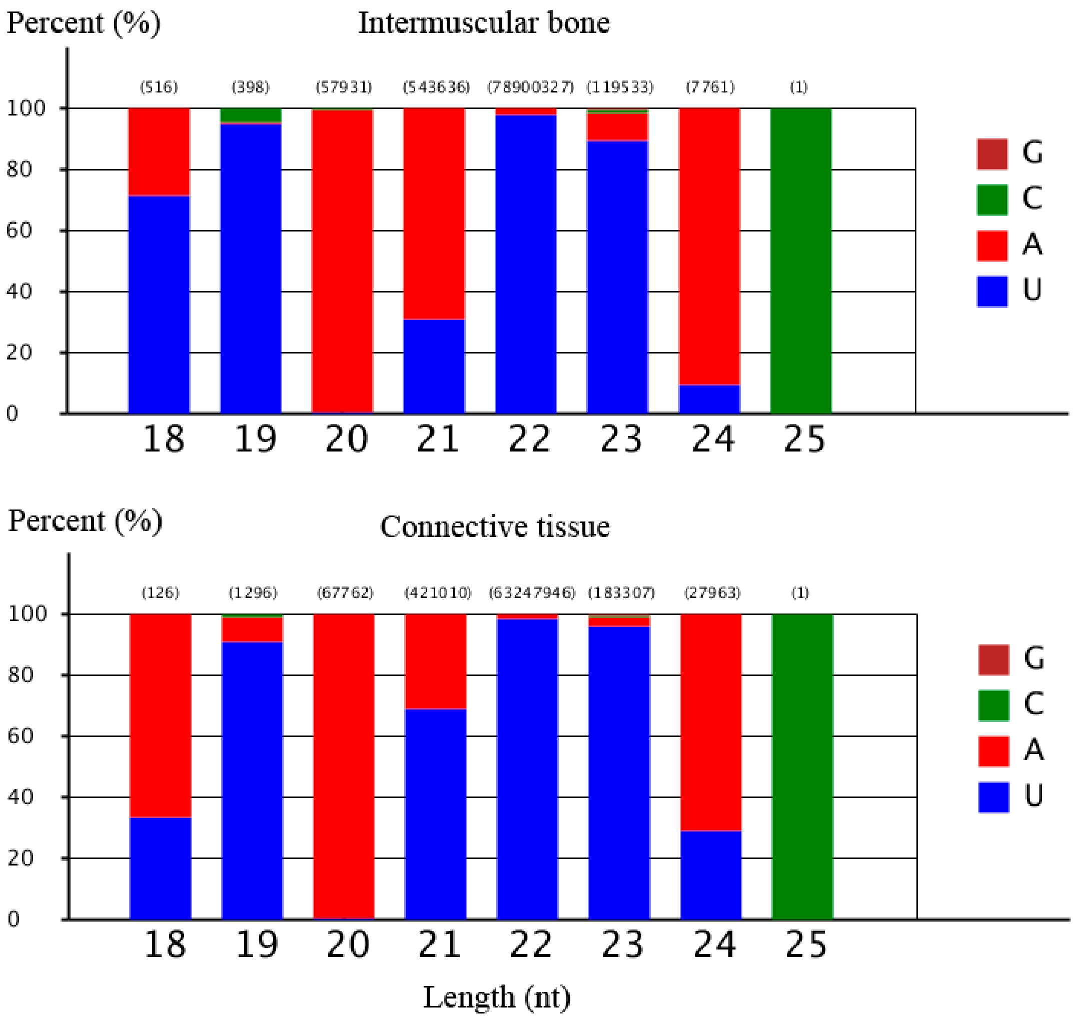


**Figure S2.** First nucleotide bias of 18~25 nt sRNA tags. The numbers above the histogram stand for the tags count in total. Each color in the figure shows the sRNA tags whose first base is a certain base.

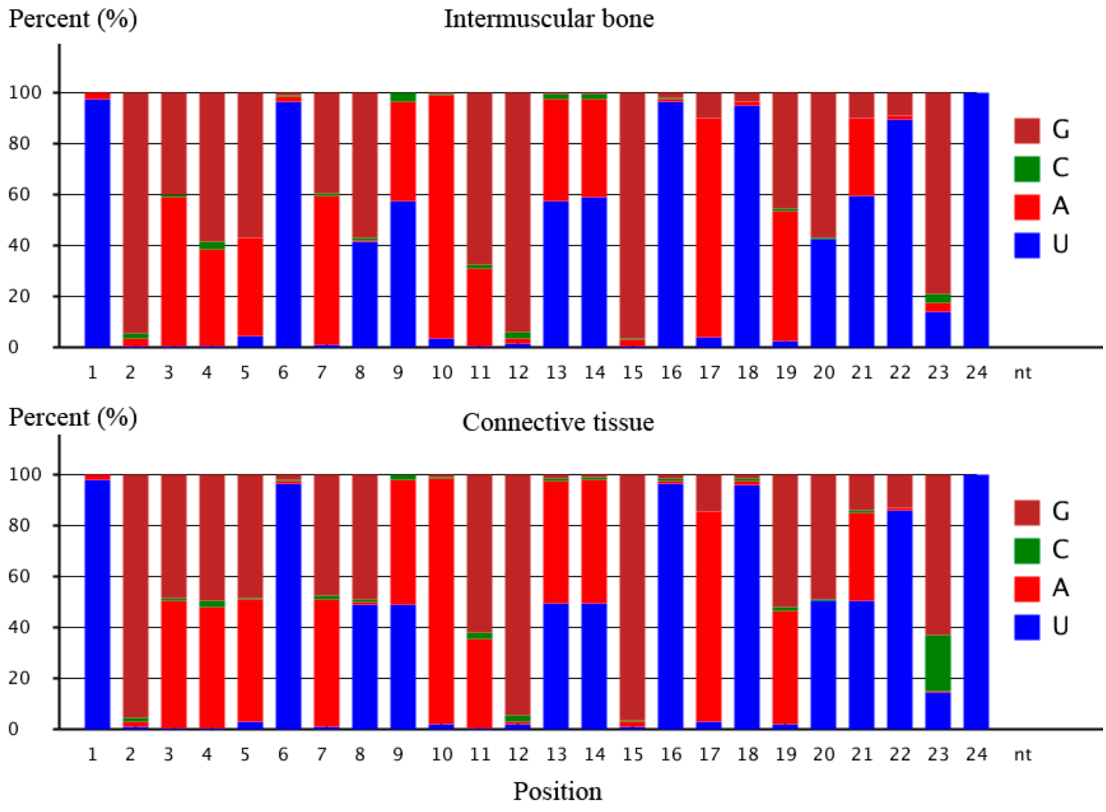


**Figure S3.** Base bias of the known miRNAs of intermuscular bone and connective tissue in *M. amblycephala* at each postion.
